# Epidemiology and economic burden of hospitalizations attributable to Respiratory Syncytial Virus (RSV) infection among infants in France, 2016 to 2023 – EPIBREATHE study

**DOI:** 10.1186/s12879-025-12039-2

**Published:** 2025-11-20

**Authors:** Robert Cohen, Marta C. Nunes, Alexis Rybak, Louis Chillotti, Céline Kernaleguen, Françoise Bugnard, William Greenwood, Yasmine Fahfouhi, Stéphane Fiévez, Emmanuelle Blanc, Corinne Levy, Marie-Laure Charkaluk

**Affiliations:** 1 Neonatology, Centre Hospitalier Intercommunal de Créteil, Créteil, France; 2 ACTIV-Association Clinique et Thérapeutique Infantile du Val de Marne, Créteil, France; 3 Center of Excellence in Respiratory Pathogens, Hospices Civils de Lyon, Lyon, France; 4 Department of Pediatrics, Department Woman-Mother-Child, Lausanne University Hospital (Centre Hospitalier Universitaire Vaudois) and University of Lausanne, Lausanne, Switzerland; 5 Stève consultants, a Cytel Company, Oullins, France; 6 Pfizer France, Paris, France; 7 Neonatology, Lille Catholic Institute Hospital Group, Lille, France

**Keywords:** Claims database, RSV, Hospital burden, Epidemiology, Infectious diseases, Pediatrics, Real-world evidence

## Abstract

**Objectives:**

Most of the clinical and economic burden of Respiratory Syncytial Virus (RSV) in pediatric populations is concentrated among infants aged < 1 year. This study aimed to estimate the recent incidence, healthcare costs and rehospitalization risk associated with RSV-related hospitalizations in infants < 1 year of age in France.

**Methods:**

The study analyzed the French National Hospital Database (PMSI) between January 1, 2016 and December 31, 2023. Outside of the classically defined epidemic season (October–March), RSV-related hospitalizations in infants were identified based on RSV-specific diagnosis codes and by both specific and non-specific RSV diagnosis codes during the epidemic season. Analyses were stratified by calendar months, by age in months and weeks for infants under 2 months. Lengths of stays (LOS), intensive care units (ICU) admissions, costs and rehospitalization rates were described. Rehospitalization rate was assessed using the Kaplan-Meier estimator.

**Results:**

A total of 354,837 RSV-related hospitalizations were identified, representing an average of 44,355 per year, and a rate of 4.9 hospitalizations per 1,000 births. Overall, 76.4% occurred between November and February (epidemic peak), and 21.1% in September-October and March-April (transition months). ICU admission was observed in 27.2% of hospitalizations. The median (Q1–Q3) hospital and ICU LOS were 3.0 days (1.0–5.0) and 3.0 days (2.0–5.0), respectively. The mean (SD) hospital cost was €3,110 (€4,694), for an annual cumulative cost of €137.9 million. Younger infants had longer and costlier stays, with greater need for ICU. Infants younger than 3 months accounted for 46.5% of the hospitalizations, 58.5% of ICU stays and 54.9% of cumulative LOS. RSV-associated rehospitalization rates [95%CI] were 10.1% [10.0%–10.3%] and 11.7% [11.5%–11.9%] at three and 12 months, respectively.

**Conclusions:**

RSV infections are estimated to cause > 44,000 hospitalizations annually in infants in France, representing a total cost exceeding €130 million, annually. This study highlighted the impact of transitional months in RSV circulation. It also emphasized that the youngest patients (< 3 months) represent nearly 50% of the economic burden. To reduce RSV disease burden, it is essential to protect infants against RSV both inside and outside the classically defined epidemic season, especially in the first months of life.

**Clinical trial:**

Not applicable.

**Supplementary Information:**

The online version contains supplementary material available at 10.1186/s12879-025-12039-2.

## Introduction

RSV is a major cause of hospitalization for acute lower respiratory tract infections among children, and notably infants (i.e., from birth to 1 year of age), mainly manifesting as bronchiolitis (up to 80% of the cases) and/or pneumopathy [1–3]. Although circulating year-round, RSV infections are seasonal. In the northern hemisphere, the epidemic season is classically defined as being between October and March [4, 5].

Medical management of RSV infection consists in supportive care (hydration, nutrition and respiratory assistance), as no specific antiviral therapy is currently available [6]. Until recently, palivizumab was the only preventive option in France, but its use was restricted to high-risk infants (e.g., preterm, recent pulmonary impairment, congenital cardiopathy) [7, 8]. In 2022, the European Medicines Agency (EMA) approved nirsevimab, a long-acting monoclonal antibody targeting site Ø of the prefusion conformation of the RSV F protein [9]. It was first made available in France in September 2023, for infants in their first epidemic season. Because of supply shortages, not all infants could be immunized during the 2023–2024 season [10]. Additionally, a bivalent vaccine based on the same F protein’s conformation (RSVPreF vaccine) from RSV-A and RSV-B was approved by EMA in 2023 and used in France from September 2024 onwards. The current guidelines for the use of this vaccine in infant prophylaxis recommend the vaccination of all pregnant women reaching 32 to 36 weeks of gestational age between September and January, to protect infants through passive immunity [11].

A previous study using national health claims data estimated the incidence and economic burden of RSV-associated hospitalizations in children under five years of age in France from 2010 to 2018. It found that more than 45,000 RSV-related hospitalizations occurred each epidemic season (October to March), with infants under one year accounting for 69% of these. The corresponding economic burden was estimated at 116 million euros per season, of which almost 80% was attributable to hospitalizations in infants younger than one year [12].

Given this context, it is essential to update available epidemiological data on RSV hospital burden, particularly on the distribution of RSV-related hospitalizations by age and calendar month in infants, in order to position these different prevention strategies appropriately. The Epibreathe study aimed to provide updated data on the epidemiological and economic burden of RSV infections in France, focusing on infants younger than 1 year of age, who face the highest disease burden, by (i) analyzing the number of RSV-related hospitalizations by month of age and calendar month from 2016 to 2023; (ii) describing the main healthcare resource use (HCRU) and associated costs during these hospitalizations; and (iii) assessing the risk of rehospitalizations, during the 2020–2023 period.

Methods.

## Study design

This observational retrospective study was conducted using data from the French National Hospital Database (*Programme de Médicalisation des Systèmes d’Information* [PMSI-MCO]). Data available include patient characteristics (e.g., age, sex) and information on hospitalization (e.g., admission date and length of stay [LOS]; main, secondary, and associated diagnoses; medical units; admission origin and discharge destination; hospitalization costs). This database is exhaustive on all hospitalizations in France, both in public and private hospitals [13].

### Selection criteria

All RSV-related hospitalizations that occurred between January 1, 2016, and December 31, 2023, among infants under one year of age were included. RSV-related hospitalizations were identified using the International Classification of Diseases, 10th Revision (ICD-10) diagnosis codes. RSV lower respiratory tract infection-specific codes (J121, J205, J210) were considered regardless of the calendar month, while non-specific codes (B974, J208, J209, J218, J219, J45) were only included during the epidemic season (October to March) (Supplementary Fig. 1 and supplementary Table 1). Two sensitivity analyses, modifying the use of non-specific codes were done and compared to data from literature: the conservative scenario only considered RSV-specific codes, while the annual scenario considered both specific and non-specific codes, whatever the calendar month [12, 14, 15].

## Outcomes and analyses

The primary outcomes were the number of RSV-related hospitalizations and the number of patients involved. The secondary outcomes included the characteristics of the RSV-related hospitalizations and HCRU, including admission and discharge modalities, diagnosis-related group (DRG), intensive care unit (ICU) admissions (see Supplementary Table 2), length of stay (LOS – both in hospital and ICU), associated costs, and the risk of rehospitalization.

The number of stays and patients were described overall, by calendar month and year. The number of stays were described across three distinct periods: before the COVID-19 pandemic (2016–2019), during the pandemic (2020–2021), and after the pandemic (2022–2023). RSV hospitalization rates were calculated by dividing the number of RSV-related hospitalizations for each combination of month-year and infant’s age in months by the estimated number of infants at risk based on French census [16] (e.g., the number of hospitalizations in January 2023 among infants aged 2 months was divided by the number of live births in November 2022). Results were then averaged across the study period, both overall and by calendar month. Sociodemographic characteristics (e.g., age, sex) and clinical characteristics (assessed between birth and RSV-related hospitalization – preterm status, cardiopathy, bronchopulmonary dysplasia, cystic fibrosis, Down syndrome – see Supplementary Tables 3 and 4) were described at the time of hospital admission. Preterm status was defined based on data from birth hospitalizations.

Costs were assessed from the perspective of the French national Health insurance i.e., direct hospitalization costs reimbursed by the French insurance). Hospitalization costs available include the DRG-related cost and supplemental costs. DRG cost corresponds to the estimated cost of a standard stay for a given disease management, weighted by severity (including costs from medical and paramedical care, usual drugs and medical devices, hospital functioning costs…). Supplemental costs correspond to care management billed in addition to the DRG cost, notably involving expensive drugs and medical devices, expensive surgical procedures, and stays in specific wards such as ICU. For the present study, as ICU represented almost all supplemental costs, only these were accounted for [17]. All costs were adjusted for inflation and reported in 2023 euros [18]. Of note, direct cost for the patient in the public French healthcare system are very low, and cannot be identified within this database, since they are outside of the national insurance perspective.

The risk of rehospitalization was assessed among infants who experienced a first RSV-related hospitalization between January 1, 2020, and December 31, 2023 (see Supplementary Table 5). This is due to the date format in PMSI-MCO before this date (month-year only). Rehospitalization-free survival function was estimated using the Kaplan-Meier (KM) estimator, with separated analyses conducted for all-causes, respiratory-related (defined as any hospitalization with an ICD-10 code from chapter X: Diseases of the respiratory system), and RSV-related hospitalizations. Rehospitalization rates were described as percentages with 95% confidence intervals (95% CI), and KM curves were displayed to reflect cumulative rehospitalization risk over time.

Continuous variables were summarized using means, standard deviations (SD), medians, and quartile ranges (Q1–Q3). Categorical variables were described as absolute and relative frequencies. Analyses were conducted using SAS statistical software (version 9.4 or later; SAS Institute Inc., Cary, NC, US). Analyses were stratified by age in months, by age group ([0–3[ months, [3–6[ months, [6–12[ months) and by age in weeks for infants younger than 2 months. Each year was further categorized into epidemic seasons (in-season (October-March)/off-season) and three four-month viral circulation periods (epidemic peak [November-February], transition period [September-October and March-April], and low circulation period [May-September]).

This study was carried out in compliance with French regulations on access to the PMSI database, for which informed consent is not required, and was conducted under the MR-006 regulatory process [19, 20].

## Results

### RSV-related hospitalizations and seasonality

Between 2016 and 2023, a total of 354,837 RSV-related hospitalizations were identified, involving 296,903 infants hospitalized during the study period, corresponding to an average of 44,355 hospitalizations per year and an average of 1.2 hospitalizations per patient. The overall hospitalization rate of RSV-related hospitalizations was 4.9 hospitalizations per 1,000 births in this population (Table 1). During this period, hospitalizations were lowest in August (*n* = 159) and peaked in December (*n* = 12,610), followed by a progressive decline until July. The classically defined RSV epidemic season (October to March) accounted for 95.5% (*n* = 338,861) of hospitalizations (Table 1). Sensitivity analyses on non-specific codes showed significant differences between scenarios and highlighted that the main scenario considering non-specific codes during epidemic season only was the closest to data from the literature. Results are available in Supplementary Tables 6 and 7.

**Table 1:** Number of hospitalizations and patient characteristics by age group (2016–2023)

Number of hospitalizations				Age			
		[0–1] month (n=43,145)			[2-3] months	[3–6] months	Overall
		[0-1] week (n=1,796)	[1-2] weeks (n=10,751)	[2-4] weeks (n=30,598)	(n=50,814)	(n=98,498)	(N=354,837)
**Annual incidence of hospitalizations**							
Average absolute number,n (%)		225 (0.5%)	1,344 (3.0%)	3,825 (8.6%)	6,352 (14.3%)	12,312 (27.8%)	44,355 (100.0%)
Incidence rate/1,000 births (SD)		7.3 (8.4)			8.3 (9.5)	5.3 (5.9)	4.9 (6.9)
**Number of hospitalizations by year, n (%)** ^**a**^							
2016		178 (0.4%)	1,111 (2.7%)	3,337 (8.2%)	5,887 (14.5%)	10,998 (27.0%)	40,669 (100.0%)
2017		226 (0.5%)	1,401 (2.9%)	4,088 (8.6%)	6,933 (14.6%)	12,888 (27.1%)	47,573 (100.0%)
2018		179 (0.4%)	1,457 (3.2%)	3,902 (8.7%)	6,569 (14.6%)	12,118 (26.9%)	44,978 (100.0%)
2019		215 (0.5%)	1,576 (3.4%)	4,212 (9%)	6,829 (14.5%)	13,061 (27.8%)	46,967 (100.0%)
2020		99 (0.5%)	619 (2.9%)	1,670 (7.8%)	2,930 (13.7%)	5,896 (27.5%)	21,425 (100.0%)
2021		157 (0.3%)	1,654 (3.1%)	5,095 (9.7%)	7,734 (14.7%)	14,056 (26.7%)	52,663 (100.0%)
2022		210 (0.4%)	1,894 (3.3%)	5,363 (9.4%)	8,336 (14.6%)	16,081 (28.1%)	57,171 (100.0%)
2023		532 (1.2%)	1,039 (2.4%)	2,931 (6.8%)	5,596 (12.9%)	13,400 (30.9%)	43,391 (100.0%)
**Number of hospitalizations by month, n (%)** ^b1^							
January		248 (13.8%)	2,099 (19.5%)	5,633 (18.4%)	9,771 (19.2%)	16,806 (17.1%)	62,381 (17.6%)
February		157 (8.7%)	1,029 (9.6%)	2,924 (9.6%)	5,347 (10.5%)	10,937 (11.1%)	38,815 (10.9%)
March		124 (6.9%)	600 (5.6%)	1,932 (6.3%)	3,481 (6.9%)	8,191 (8.3%)	29,173 (8.2%)
April		20 (1.1%)	199 (1.9%)	574 (1.9%)	727 (1.4%)	1,266 (1.3%)	4,760 (1.3%)
May		20 (1.1%)	93 (0.9%)	234 (0.8%)	361 (0.7%)	544 (0.6%)	2,124 (0.6%)
June		19 (1.1%)	77 (0.7%)	216 (0.7%)	381 (0.7%)	679 (0.7%)	2,400 (0.7%)
July		19 (1.1%)	136 (1.3%)	315 (1%)	497 (1%)	783 (0.8%)	3,109 (0.9%)
August		13 (0.7%)	63 (0.6%)	149 (0.5%)	196 (0.4%)	346 (0.4%)	1,274 (0.4%)
September		34 (1.9%)	113 (1.1%)	220 (0.7%)	328 (0.6%)	671 (0.7%)	2,309 (0.7%)
October		343 (19.1%)	1,073 (10%)	3,145 (10.3%)	5,153 (1.1%)	10,629 (10.8%)	38,490 (10.8%)
November		361 (20.1%)	2,019 (18.8%)	5,954 (19.5%)	9,756 (19.2%)	19,557 (19.9%)	69,119 (19.5%)
December		438 (24.4%)	3,250 (30.2%)	9,302 (30.4%)	14,816 (29.2%)	28,089 (28.5%)	100,883 (28.4%)
Total		1,796 (100%)	10,751 (100%)	30,598 (100%)	50,814 (100%)	98,498 (100.0%)	354,837 (100.0%)
**Number of hospitalizations per periods, n (%)** ^b2^							
Epidemic peak (NOV-FEB)		1204 (67.0%)	8397 (78,1%)	23813 (77,8%)	39690 (78,1%)	75389 (76,5%)	271198 (76,4%)
Transition periods (SEP-OCT / MAR-APR)		521 (29.0%)	1985 (18,5%)	5871 (19,2%)	9689 (19,1%)	20757 (21,1%)	74732 (21,1%)
Low circulation (MAY-AUG)		71 (4.0%)	369 (3,4%)	914 (3%)	1435 (2,8%)	2352 (2,4%)	8907 (2,5%)
**Term, n (%)** ^b3,c^							
Full term		35,390 (82.0%)			36,724 (72.3%)	69,170 (70.2%)	256,891 (72.4%)
Preterm		944 (2.2%)			3,480 (6.8%)	8,130 (8.3%)	23,745 (6.7%)
Not defined ^d^		6,811 (15.8%)			10,610 (20.9%)	21,298 (21.5%)	74,201 (20.9%)

^a^Percentages calculated by age class;

^b^Percentages calculated by month (1), period (2), term (3); ^c^Term defined according to data from birth hospitalizations; ^d^Corresponds to cases with missing data

Note: for each age category, the bound displayed includes the lowest age in the group (i.e., “[“)and excludes the highest age (i.e. “[“). For instance, for the [0-1[ month group, patients included are aged between 0 and less than 1 month; while patients aged 1 month or older will be included in the subsequent category

From 2016 to 2019, before the COVID-19 pandemic, the average annual number of RSV-related hospitalizations was 45,047. During this period, the seasonal pattern was stable, with peaks in the winter, moderate circulation in spring and fall, and almost no circulation in the summer. The pandemic disrupted the usual RSV seasonal pattern. In 2020, the number of RSV-related hospitalizations dropped by almost 50% compared to the 2016–2019 average, falling from 45,047 to 21,425 cases with an incidence of 2.4 hospitalizations per 1,000 births. Fewer than 1,500 monthly cases were observed between April and December 2020. In 2021, towards the end of the pandemic, a resurgence in RSV cases was observed, along with a notable shift in seasonality, with 18.4% of hospitalizations occurring outside the classically defined epidemic season (October to March). The pre-pandemic seasonal pattern began to re-emerge in October 2021 with a near-complete normalization in 2022–2023(Supplementary Fig. 2). Of note, when excluding the COVID-19 period from the assessment of RSV-related hospitalization rate, this rate increased from 4.9 to 5.3 hospitalizations per 1,000 births, emphasizing the significant impact of the pandemic on RSV circulation.

**Fig. 1:**
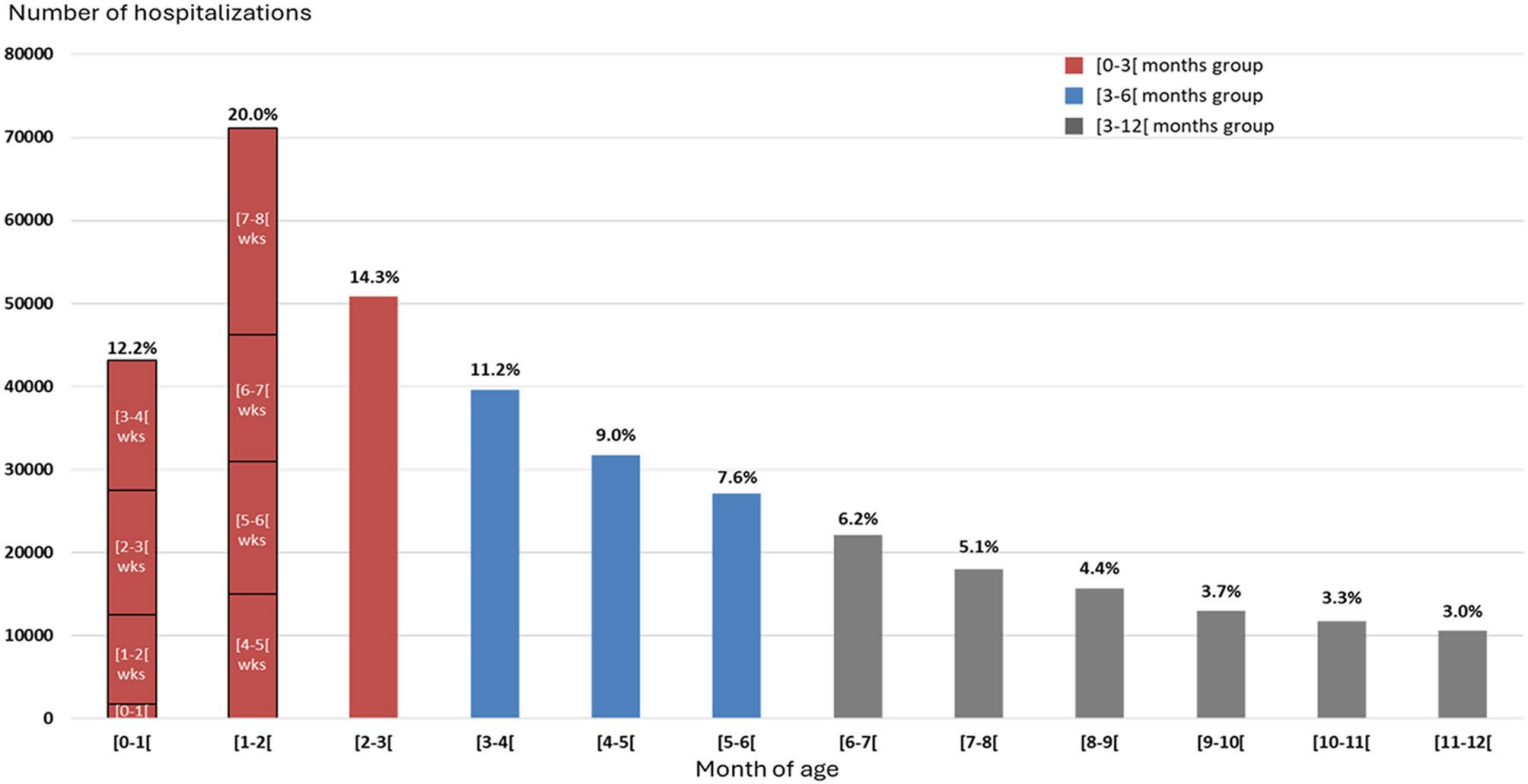
Distribution of RSV hospitalizations by month of age (by week for [0–1[ month and [1–2[ months) – study population (*N* = 354,837). For the first two months,bars are split by week of age. Note: for each age group, the bound displayedinclude the lowest age in the group and excludes the highest age. For instance,for the [0-1[ month group, patients included are aged between 0 and less than 1month; while patients aged 1 month or older will be included in the following group

Based on the monthly distribution of hospitalizations outside the pandemic period, each year was divided into three four-month periods. The low circulation period (May to August) accounted for 8,907 hospitalizations (2.5% of the total). The epidemic peak (November to February) represented the majority of cases, with 271,198 stays (76.4% of the total). The two transitional periods – March to April and September to October – accounted for 74,732 hospitalizations, representing 21.1% of the total (Table 1; Fig. 1).

## Patient characteristics

The median (Q1–Q3) age at hospitalization was 3.3 months (1.6–6.1). Infants under three months accounted for 46,5% (*n* = 165,078) of the hospitalizations, with approximately 20% (*n* = 71,119) aged between 1 and 2 months (Fig. 1). Infants younger than 6 months accounted for nearly three quarters (74.3%, *n* = 263,576) of RSV-related hospitalizations (Fig. 1). The analysis by age in weeks among patients less than two months (*n* = 119,943) showed that 1.5% (*n* = 1,796) of infants were hospitalized during their first week of life. Weekly hospitalization counts were much higher and remained stable between week 2 and week 7 of life, each accounting for roughly around 13% of the hospitalizations each week and peaked at week 8 with 22.4% (*n* = 26,842) of hospitalizations (Table 1).

Male infants accounted for 57.9% (*n* = 205,594) of hospitalizations, representing a sex-ratio of 1.4. Prematurity status was available for 280,636 (79.1%) cases, among which 23,745 (8.5%) involved preterm infants.

**Fig. 2:**
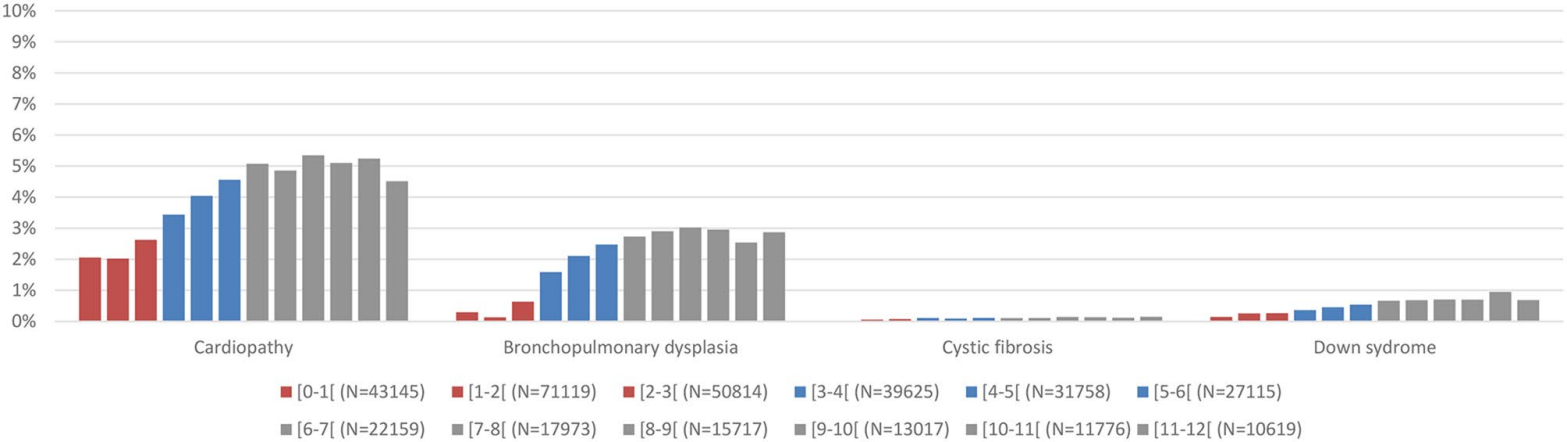
Distribution of comorbidities of interest by month of age. Note: for each age group, the bound displayed include the lowest age in the group and excludes the highest age. For instance, for the [0–1[ month group, patients included are aged between 0 and less than 1 month; while patients aged 1 month or older will be included in the following group

Overall, 4.4% of hospitalizations involved at least one risk factor of interest other than prematurity. This proportion tended to increase with age, from 2.3% in the first month, to approximately 7% in infants aged 10 to 12 months (Fig. 2). No notable differences were observed in patient characteristics between hospitalizations occurring during in-season and off-season periods.

The most frequent risk factors were congenital cardiopathy (3.4%) and bronchopulmonary dysplasia (1.4%), while cystic fibrosis and Down syndrome each accounted for less than 0.5% of cases.

### Inpatient HCRU

Hospitalizations were almost exclusively (99.6%) done in public hospitals. Most hospitalizations involved an admission via emergency room (82.3% of hospitalizations, *n* = 291,973), while 12.6% (*n* = 44,558) involved a direct admission from home. The most frequent discharge modality was home (93.4%, *n* = 331,468), followed by inter-hospital transfers (6.6%, *n* = 23,222). a total of 174 inpatient deaths occurred during the study period.

Overall, 27.2% (*n* = 96,585) of RSV-related hospitalizations required a stay in the ICU. This rate was highest among infants younger than 1 month (44.5%, *n* = 19,204) and progressively decreased with age to 17.3% (*n* = 1,838) among 12-month-olds. The overall median (Q1–Q3) hospital LOS (including ICU when appropriate) was 3.0 days (1.0–5.0). Median ICU LOS was also 3.0 days (2.0–5.0). Median hospital LOS decreased from 4.0 days (2.0–7.0) in infants aged less than 1 month, to 2.0 days (1.0–4.0) among 12-month-olds. Median ICU LOS followed a similar pattern, decreasing from 4.0 days (2.0–7.0) to 3.0 days (2.0 to– 4.0), respectively. Infants younger than 3 months accounted for the majority of hospital-related resource use, representing 58.5% (*n* = 56,479) of ICU stays, 54.9% of cumulative hospital LOS, and 63.7% of cumulative ICU LOS. For infants younger than 6 months, these rates were 82.3% of ICU stays, 79.3% of hospital LOS, and 84.7% of ICU LOS (Table 2).

**Table 2:** Description of hospitalizations and economic results

	Age							
	[0–1] month (n=43,145)		[1-2] months		[2-3] months	[3–6] months	[6–12] months	Overall
	[0-1] week (n=1,796)	[1-2] weeks (n=10,751)	[2-4] weeks	(n=71,119) (n=30,598)	(n=50,814)	(n=98,498)	(n=91,261)	(N=354,837)
Number of admissions, n (%)								
Hospitalizations	1,796 (0.5%)	10,751 (3.0%)	30,598 (8.6%)	71,119 (20.0%)	50,814 (14.3%)	98,498 (27.8%)	91,261 (25.7%)	354,837 (100.0%)
ICU stays^a^	981 (54.6%)	5,600 (52.1%)	12,623 (41.3%)	22,969 (32.3%)	14,306 (28.2%)	23,071 (23.4%)	17,035 (18.7%)	96,585 (27.2%)
Hospital LOS								
Median (Q1-Q3)	10.0 (4.0–30.0)	6.0 (3.0–9.0)	4.0 (2.0–7.0)	3.0 (2.0 - 5.0)	3.0 (2.0 - 5.0)	3.0 (1.0–4.0)	2.0 (1.0–4.0)	3.0 (1.0–5.0)
ICU LOS								
Median (Q1-Q3)	12.0 (4.0–40.0)	5.0 (3.0–8.0)	4.0 (2.0 - 6.0)	4.0 (2.0–5.0)	3.0 (2.0–5.0)	3.0 (2.0–5.0)	3.0 (2.0–5.0)	3.0 (2.0–5.0)
Cumulative length of stay, days (%)^b^								
Hospitalizations	50,237 (3.7%)	66,945 (5.0%)	141,925 (10.6%)	281,174 (20.9%)	190,852 (14.2%)	338,839 (25.2%)	278,407 (20.8%)	1,348,381 (100.0%)
ICU stays	31,061 (6.8%)	34,120 (7.5%)	61,218 (13.5%)	100,944 (22.2%)	61,443 (13.5%)	96,066 (21.2%)	69,099 (15.2%)	453,950 (100.0%)
Hospital costs per infant, €, mean (SD)								
Mean (SD)	€3,528 (5,999)		€3,148 (5,353)		€3,114 (5,635)	€3,097 (5,266)	€2,902 (3,773)	€3,110 (4,694)
Distribution of mean hospital costs (%)								
	74.40%		76.90%		75.80%	75.40%	73.30%	75.10%
	25.60%		23.10%		24.20%	24.60%	26.70%	24.90%
Annual cumulative hospital costs, € (%)^b^								
Average annual total costs	€19,022,536 (13.8%)		€27,988,223 (20.3%)		€19,674,213 (14.3%)	€38,136,550 (27.6%)	€33,109,794 (24.0%)	€137,931,377 (100.0%)

^a^ ICU stays among hospitalizations correspond to the number of ICU stays divided by the number of hospitalizations by age group

^b^ percentages correspond to the cumulative LOS/cost in the age group, divided by the overall cumulative LOS/cost

Note: for each age group, the bound displayed include the lowest age in the group and excludes the highest age. For instance, for the [0-1[ month group, patients included are aged between 0 and less than 1 month; while patients aged 1 month or older will be included in the following group

Analysis by age in weeks showed a gradual decrease in ICU utilization. Among infants hospitalized in their first week of life, 54.6% required ICU admission, compared with 30.2% by the eighth week. The median (Q1–Q3) hospital LOS decreased from 10.0 days (4.0–30.0) in the first week to 3.0 days (2.0–5.0) in 8-week-old infants. The median (Q1–Q3) ICU LOS also decreased from 12.0 days (4.0–40.0) to 3.0 days (2.0–5.0) for the same age groups, respectively (Table 2).

## Hospitalization costs

**Fig. 3:**
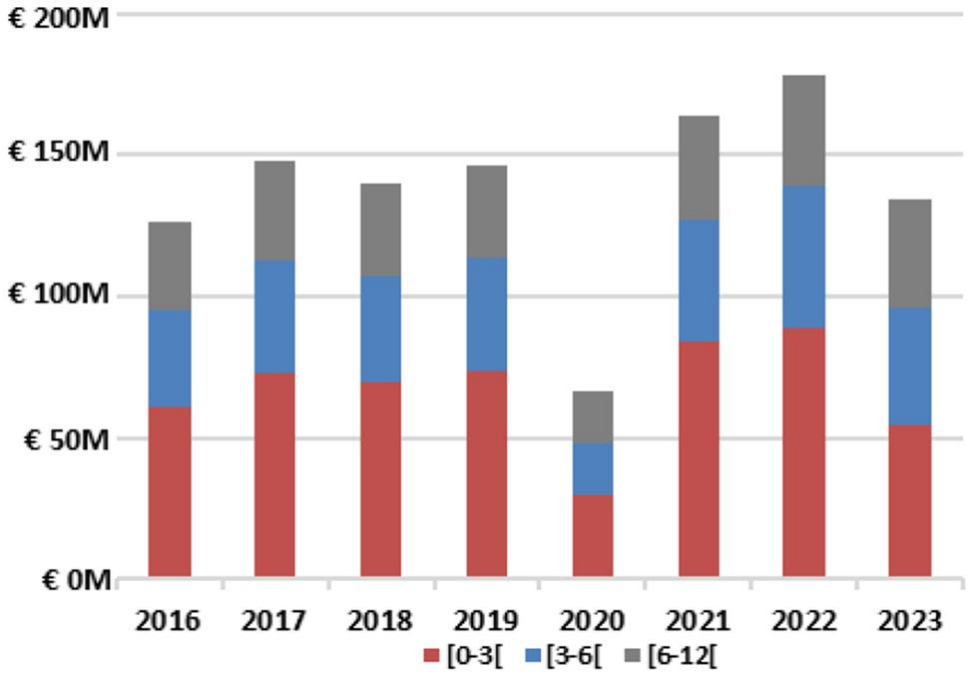
Annual cumulative costs of RSV-related hospitalizations by age groups. Note: for each age group, the bound displayed include the lowest age in the group and excludes the highest age. For instance, for the [0-3[ month group, patients included are aged between 0 and less than 3 months; while patients aged 3 months or older will be included in the following group

The mean (SD) cost of an RSV-related hospitalization was €3,110 (€4,694), mostly driven by hospitalization stay fees (diagnosis-related group), which represented around 75% of the total costs. The cost distribution was similar across age groups, although total costs tended to decrease with age, from €3,232 (€4,861) among infants younger than 3 months, to €2,902 (€3,773) for those aged between 6 and 12 months (Table 2). The total economic burden of RSV-related hospitalizations among infants was estimated at approximately €138 million per year. When excluding the pandemic year (2020), this figure increased to €150 million annually. Almost half (48.3%) of these cumulative costs were attributed to hospitalizations of infants younger than 3 months, while this rate was 76.0% when considering hospitalizations of infants younger than 6 months (Fig. 3).

## Rehospitalizations

Among the 296,903 infants hospitalized during the study period, 49.3% (*n* = 146,481) had their first RSV-related hospitalization between January 1, 2020, and December 31, 2023, and were followed to assess the risk of rehospitalization.

**Fig. 4:**
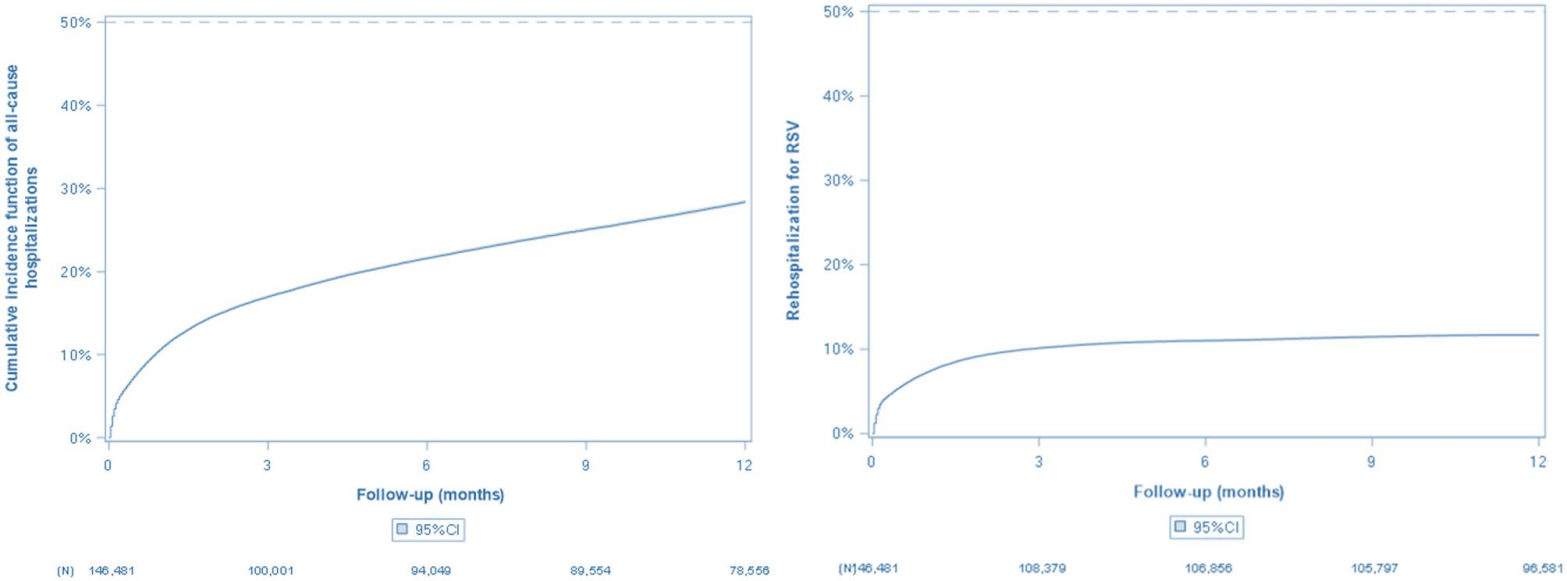
Rehospitalization rates by Kaplan-Meier estimator: (**A**) all-cause; (**B**) RSV-specific

The rate [95%CI] of infants rehospitalized for any cause after their initial RSV-related hospitalization increased from 10.7% [10.6%–10.9%] at one month to 28.4% [28.1%–28.6%] at one year post-discharge. For respiratory-related rehospitalizations (including RSV), rates increased from 8.1% [7.9%–8.2%] at one month to 18.1% [17.9%–18.4%] at one year, respectively. RSV-specific rehospitalization rate [95%CI] was 7.3% [7.2%–7.4%] at one month and plateaued after three months, reaching 10.1% [10.0%–10.3%] at three months, and 11.7% [11.5%–11.9%] at one year, respectively (Fig. 4).

## Discussion

This study provided an updated and detailed overview of the epidemiology and economic burden of RSV-related hospitalizations in infants younger than 1 year of age in France, as well as the risk of rehospitalizations. To our knowledge, this is the first French real-world study to assess RSV-related hospital burden among this population with stratification both by calendar month and infant age in weeks and months. Furthermore, it introduced an alternative framework for defining RSV circulation periods in claims databases, distinguishing between the epidemic peak and seasonal transitions, which may better inform the timing of prevention strategies.

On average, approximately 44,000 RSV-related hospitalizations occurred per year. This corresponds to an overall incidence of 4.9 cases per 1,000 births (or 5.3 when excluding the COVID-19 period), mainly involving the youngest infants. These results are consistent with recent European claims-based studies which used comparable algorithms for the identification of the RSV cases. Demont et al. (2021) reported an average of 35,000 hospitalizations per epidemic seasons in France, with a similar algorithm [12]. Similarly, the study by Martinón-Torres et al. (2023) [21], conducted in Spain, reported that 57.5% of RSV-related hospitalizations among infants involved those younger than 3 months; a similar percentage (50.1%) was reported for the same age group in Portugal by Bandeira et al. (2023) [22]. The similarities in these findings highlight the significant impact of RSV-related hospitalizations in the first months of life and demonstrate the transposability of RSV studies across European countries in terms of epidemiology and patient characteristics.

This study also allowed for a more granular understanding of RSV seasonal dynamics by describing the distribution of cases by calendar month. While RSV circulation is typically examined across two periods – during and outside of the epidemic season – our findings support a three-period framework: an epidemic peak period (November to February), a low-circulation period (May to August), and two transitional periods (March to April and September to October). The epidemic peak alone accounted for approximately 75% of the hospitalizations, while transitional months represented another 20% - a proportion too large to ignore in the planning of preventive measures. This further stratification could play a pivotal role in optimizing the definition of circulation periods, during which prophylactic programs could be implemented. This temporal pattern should be considered when aligning immunization schedules with periods of risk. For example, the current guidelines for maternal RSVPreF vaccination in France recommend immunizing pregnant women between 32 and 36 weeks of gestational age from September to January, and leading to a partial protection of infants born during transition periods, notably before the epidemic peak [23].

The observed concentration of more than 95% of hospitalizations during the classically defined epidemic season (October to March) is partly explained by the codes and algorithms used to identify RSV-related hospitalizations. While RSV-specific codes were searched for year-round, RSV non-specific codes were only identified during the epidemic season, in order to account for the possible difficulties obtaining a formal RSV diagnosis during the peak season [24, 25].

Bearing in mind the specificities of neonatal care, the results of this study indicated that the youngest patients tended to require more frequent, more intensive, and more expensive hospital stays. Almost 50% of all RSV-related hospitalizations occurred in infants patients younger than 3 months, and 75% in those under 6 months, in line with data from the literature [26–28]. The youngest infants also had the longest hospital stays (both overall and in ICU), more frequent ICU admissions, and highest related costs. Longer LOS among the youngest patients were also observed in studies conducted in Spain and Portugal (up to 7.9 days) and in Japan (up to 7.5 days) [21, 22, 29]. In the Portuguese study, a decreasing trend was also identified – from 7.5 days among infants younger than 1 month to 4.9 days among infants older than 6 months [22]. This trend was identified for both ICU stays and overall costs. These results reflect the overall vulnerability of infants, the ongoing pulmonary development and the progressive maturation of the newborn’s immune system, which almost exclusively relies on maternal antibodies transferred from the mother via the placenta or breast milk during the first months of life [30–32].

This trend was even more apparent in the analysis by week of age, with a peak in both ICU use (54.6%) and LOS (10 days in hospital, 12 days in ICU in median) among infants aged less than one week. Of note, the proportion of patients aged less than one week was relatively low due to the lower risk of exposure, the strong hygiene, the incubation period and the potential maternal antibodies transferred to the newborn.

The risk of rehospitalizations within one year was also assessed for approximately 150,000 infants with a first RSV-related hospitalization between 2020 and 2023. More than 10% of these infants had been rehospitalized within one month for any cause, and 30% within one year. These results are in line with the findings of Sarna et al. (2024), that estimated 30-day all-cause rehospitalization rates at 10.6% and 11.2% for infants aged 0–6 and 6–12 months, respectively [33]. Lee et al. (2025) described an incidence of new hospitalization for lower respiratory tract infection of 0.27 per patient-year [34]. Regarding RSV-specific rehospitalization rate, a plateau was observed after three months, with rates of 10% and 11% at three and 12 months, respectively. Two explanations may account for these observations. First, the seasonal circulation of RSV implies that infants are at the highest risk for exposure, hospitalization, and rehospitalization during the epidemic peak. Since most patients are hospitalized between November and February, the following three months span the epidemic peak and transitional periods; therefore, RSV rehospitalizations after this period are rare due to the lower circulation of RSV. In addition, as infants grow, the development of their pulmonary and immune systems may help reduce the risk of RSV-related hospitalization. Another possible explanation is linked to organizational challenges and is harder to assess. Although shorter stays are usually associated with lower severity and lower risk of rehospitalization [34], the constraints on hospitals during epidemic peaks may lead to early discharges of some lower-risk patients who have not sufficiently recovered, potentially increasing the risk of readmission for the same RSV infection [35, 36]. Finally, a risk of nosocomial infections with other bacteria and viruses (e.g., rotavirus) could lead to longer hospitalizations and early readmissions [37]. Similarly, bronchial hyperreactivity secondary to a RSV infection could lead to reinfections or infant asthma in the months following the hospitalization [38, 39].

This study leveraged the strength of PMSI, a nationwide administrative database that enables robust assessment of the epidemiology, management, and costs of hospitalization due infectious diseases such as RSV, providing exhaustive data throughout France. However, some limitations should be acknowledged. The PMSI-MCO does not include outpatient data, making it impossible to capture RSV infections solely managed in emergency rooms (estimated additional 1.2 million euros [40]) or outside the hospital setting. Infections managed only in outpatient facilities are the least severe, requiring little to no treatment. While this study focuses appropriately on more severe cases requiring hospitalization, broader surveillance strategies will be needed to understand the full burden of RSV, including mild and moderate cases. This is also the case for indirect costs notably due to parental leaves, which can significantly increase the overall burden of RSV infections. A recent French study estimated an average of two days of unpaid leave to take care of a child with RSV infection managed in primary care [41].

Real-world data are susceptible to misclassification, and potential under- or mis-reporting of diagnoses resulting in a risk of information bias, even if minimal [42, 43]. Another limitation could be the presence of the ICD-10 code J21.9 (“acute bronchiolitis, unspecified”) which, while frequently used, is not specific to RSV infections (almost 28,000 (40%) stays had a J21.9 code in 2019 [44]). This could lead to a decrease in specificity and the inclusion of patients with non-RSV pulmonary infections. Coding practices may depend on the seasonality of RSV infections, as biological analyses are expected not to be performed exhaustively during epidemic peaks, leading to a sizable proportion of diagnoses based on symptoms alone. Several recent studies have evaluated the performance of different coding algorithms by comparing them with RSV viral test results. Their findings showed that when used alone, RSV-specific codes have near-perfect specificity but a very low sensitivity, indicating that a significant proportion of RSV-related hospitalizations are coded otherwise. Also, non-specific codes should be used only during period of high viral circulation. However, no tested algorithms seemed to achieve both high sensitivity and specificity at the same time, most of them lacking sensitivity [45–47]. For example, Cai et al., estimated that the algorithm using RSV specific codes coupled with ICD-10 codes J12, J18, J20, J21 and J22 during epidemic seasons, reached 90% specificity, but only 40% sensitivity among children less than five years of age [47]. These findings are expected to drastically change in the most recent years, thanks to the generalization of multiplex viral testing methods, allowing for the detection of the main respiratory viruses, notably influenza, COVID-19, and RSV [48, 49].

Our conservative approach, limiting non-specific codes to the epidemic season (October-March) – takes into consideration this coding practice. According to Meissner et al. (2016) up to 80% of bronchiolitis cases were attributable to RSV among children, limiting the impact of this bias [2]. Finally, due to the COVID-19 pandemic, the 2020 data were not representative of the current RSV epidemiology [50], as COVID-19 itself and the resulting public health restrictions drastically changed the RSV seasonal pattern [51–54]. While these data were included for completeness, they should not be considered representative of long-term trends.

## Conclusion

An estimated 44,000 hospitalizations are caused by RSV infections each year, leading to an annual economic burden of more than €130 million. While most RSV-related hospitalizations occurred between November and February, the transitional periods (March to April and September to October) accounted for a significant part of RSV epidemiology. These data provide a solid baseline before RSV-prophylaxis implementation in France and this extended burden pleads in favor of an equally broad immunization campaign. In addition, this study highlights the extent to which the burden of disease is concentrated in the youngest infants. The first three months of life accounted for almost 50% of all stays and the first six months for 75%. Furthermore, the first weeks of life concentrate the longest hospital stays and the highest rates of admission to ICU. This data argues in favor of vaccinating pregnant women, in order to protect newborns from the first days of life. Lastly, about a tenth of patients who were hospitalized for RSV infection were re-hospitalized for the same reason within two months of returning home. This data underlines the crucial importance of prevention against RSV infection, to reduce the overload of hospital services, which negatively impacts the care of infants. Implementing effective prophylactic methods through maternal vaccination or passive immunization could thus significantly reduce the clinical and economic burden of RSV in infants.

## Supplementary Information

Below is the link to the electronic supplementary material.

Supplementary Material 1

## Data Availability

As per MR-006 process regulating access to PMSI data, no additional data are available. Only authorized personnel can access these data, on preidentified secured platforms, after the submission of a MR-006 dossier to the CNIL. Additional information on MR-006 process and data access are available on CNIL (https://www.cnil.fr/sites/cnil/files/atoms/files/mr-006_methodologie_de_reference-traitements_de_donnees_du_snds_et_des_rpu_interet_legitime.pdf) and Health Data Hub (HDH) websites (https://www.health-data-hub.fr/). For further information, please contact the HDH using the following link (https://www.health-data-hub.fr/contact), or contact William Greenwood william.greenwood@pfizer.com. The complete list of algorithms is available as supplementary data.

## Abbreviations

Abbreviation: Label
DRG: Diagnosis-related group
EMA: European Medicines Agency
HCRU: Healthcare resource use
ICD-10: International Classification of Diseases, 10th Revision
ICU: Intensive care unit
LOS: Length of stay
PMSI-MCO: National Hospital Database (Programme de Médicalisation des Systèmes d’Information)
Q1-Q3: First and third quartiles
RSV: Respiratory Syncytial Virus
SD: Standard deviations
95% CI: 95% Confidence interval
