# Supplementary material for: Epidemiology and economic burden of hospitalizations attributable to Respiratory Syncytial Virus (RSV) infection among infants in France, 2016 to 2023 – EPIBREATHE study
Source: BMC Infect Dis. 2025 Nov 20;25:1621. doi: 10.1186/s12879-025-12039-2 (PMC12632121; doi:10.1186/s12879-025-12039-2)
Supplement: Supplementary file 1 — Supplementary Material 1 [file 12879_2025_12039_MOESM1_ESM.docx]

# Supplementary Material

## Supplementary Tables

**Codes and algorithms used for the identification of RSV-related hospitalizations**

Supplementary Table 1: RSV-related hospitalizations

|  | RSV-specific codes | | | | RSV-potential codes | | | | |
| --- | --- | --- | --- | --- | --- | --- | --- | --- | --- |
| ICD-10 | J121 | J205 | J210 | B974 | J208 | J209 | J218 | J219 | J45 |
|  | Year-round | Year-round | Year-round | Season OCT-MAR | Season OCT-MAR | Season OCT-MAR | Season OCT-MAR | Season OCT-MAR | Season OCT-MAR |
| *J121: RSV pneumonia ; J205: Acute bronchitis due to RSV ; J210: Acute bronchiolitis due to RSV ; B974: RSV as the cause of other diseases ; J208: Acute bronchitis due to other specified organisms ; J209: Acute bronchitis, unspecified ; J218: Acute bronchiolitis due to other specified organisms ; J219: Acute bronchiolitis, unspecified ; J45: Asthma* | | | | | | | | | |

Supplementary Table 2: Intensive care units

| 01A | *Adult Resuscitation except burns* |
| --- | --- |
| 01B | *Adult Resuscitation for burns* |
| 02A | *Cardiologic intensive care* |
| 02B | *Other internsive care* |
| 05 | *Neonatology intensive care* |
| 06 | *Neonatology resuscitation* |
| 13A | *Pediatric resuscitation except burns* |
| 13B | *Pediatric resuscitation for burns* |
| 14A | *Pediatric advanced monitoring except burns* |
| 14B | *Pediatric advanced monitoring for burns* |
| 18 | *Neurovascular intensive care* |

*Translations for information only. This is a French classification with no direct equivalent outside of France.*

Supplementary Table 3: Risk factors

| Cardiopathy | Patients with at least one hospitalization with any of the following diagnosis coded as DP/DR/DAS:   \| Q20 \| Congenital malformations of cardiac chambers and connections \| \| --- \| --- \| \| Q21 \| Congenital malformations of cardiac septa \| \| Q22 \| Congenital malformations of pulmonary and tricuspid valves \| \| Q23 \| Congenital malformations of aortic and mitral valves \| \| Q24 \| Other congenital malformations of heart \| \| Q25 \| Congenital malformations of great arteries \| \| Q26 \| Congenital malformations of great veins \| |
| --- | --- | --- | --- | --- | --- | --- | --- | --- | --- | --- | --- | --- | --- | --- | --- |
| Down syndrome | Patients with at least one hospitalization with any of the following diagnosis coded as DP/DR/DAS:   \| Q90 \| Down syndrome \| \| --- \| --- \| |
| Bronchopulmonary dysplasia | Patients with at least one hospitalization with any of the following diagnosis coded as DP/DR/DAS:   \| P271 \| Bronchopulmonary dysplasia originating in the perinatal period \| \| --- \| --- \| |
| Cystic fibrosis | Patients with at least one hospitalization with any of the following diagnosis coded as DP/DR/DAS:   \| E84 \| Cystic fibrosis \| \| --- \| --- \| |

Supplementary Table 4: Preterm status:

| ICD-10 code | label |
| --- | --- |
| P07 | Disorders related to short gestation and low birth weight, not elsewhere classified |
| H351 | Retinopathy of prematurity |
| P590 | Neonatal jaundice associated with preterm delivery |
| P612 | Anemia of prematurity |
|  | |
| **DRG code** | Label |
| **15M05A** | Neonates of 3300g and gestational age of 40 weeks and assimilated (neonatal group 1), without significant problems |
| **15M05B** | Neonates of 3300g and gestational age of 40 weeks and assimilated (neonatal group 1), with other significant problem |
| **15M05C** | Neonates of 3300g and gestational age of 40 weeks and assimilated (neonatal group 1), with severe problem |
| **15M05C** | Neonates of 3300g and gestational age of 40 weeks and assimilated (neonatal group 1), with major problem |
| **15M06A** | Neonates of 2400g and gestational age of 38 weeks and assimilated (neonatal group 2), without significant problems |
| **15M06B** | Neonates of 2400g and gestational age of 38 weeks and assimilated (neonatal group 2), with other significant problem |
| **15M06C** | Neonates of 2400g and gestational age of 38 weeks and assimilated (neonatal group 2), with severe problem |
| **15M06D** | Neonates of 2400g and gestational age of 38 weeks and assimilated (neonatal group 2), with major problem |
| **15M07A** | Neonates of 2200g and gestational age of 37 weeks and assimilated (neonatal group 3), without significant problems |
| **15M07B** | Neonates of 2200g and gestational age of 37 weeks and assimilated (neonatal group 3), with other significant problem |
| **15M07C** | Neonates of 2200g and gestational age of 37 weeks and assimilated (neonatal group 3), with major or severe problem |
| **15M08A** | Neonates of 2000g and gestational age of 37 weeks and assimilated (neonatal group 4), without significant problems |
| **15M08B** | Neonates of 2000g and gestational age of 37 weeks and assimilated (neonatal group 4), with other significant problem |
| **15M08C** | Neonates of 2000g and gestational age of 37 weeks and assimilated (neonatal group 4), with major or severe problem |
| 15M09A | Neonates of 1800g and gestational age of 36 weeks and assimilated (neonatal group 5), without significant problems |
| 15M09B | Neonates of 1800g and gestational age of 36 weeks and assimilated (neonatal group 5), with other significant problem |
| 15M09C | Neonates of 1800g and gestational age of 36 weeks and assimilated (neonatal group 5), with major or severe problem |
| 15M10A | Neonates of 1700g and gestational age of 35 weeks and assimilated (neonatal group 6), without significant problems |
| 15M10B | Neonates of 1700g and gestational age of 35 weeks and assimilated (neonatal group 6), with other significant problem |
| 15M10C | Neonates of 1700g and gestational age of 35 weeks and assimilated (neonatal group 6), with major or severe problem |
| 15M11A | Neonates of 1500g and gestational age of 33 weeks and assimilated (neonatal group 7), without significant problems |
| 15M11B | Neonates of 1500g and gestational age of 33 weeks and similar (neonatal group 7), with other significant problem |
| 15M11C | Neonates of 1500g and gestational age of 33 weeks and assimilated (neonatal group 7), with major or severe problem |
| 15M12A | Neonates of 1300g and gestational age of 32 weeks and assimilated (neonatal group 8), without significant problems |
| 15M12B | Neonates of 1300g and gestational age of 32 WA and assimilated (neonatal group 8), with significant problem |
| 15M13A | Neonates of 1100g and gestational age of 30 weeks and assimilated (neonatal group 9), without significant problems |
| 15M13B | Neonates of 1100g and gestational age of 30 weeks and assimilated (neonatal group 9), with significant problem |
| 15M14A | Neonates of 800g and gestational age of 28 weeks and assimilated (neonatal group 10), without significant problems |
| 15M14B | Neonates of 800g and gestational age of 28 weeks and assimilated (neonatal group 10), with significant problem |

*Translations for information only. This is a French classification with no direct equivalent outside of France.*

Supplementary Table 5: Rehospitalizations

| All cause rehospitalization | All hospitalizations in PMSI MCO, whatever the diagnosis |
| --- | --- |
| Rehospitalization for respiratory indication | All hospitalizations in PMSI with any of the following diagnosis coded as DP/DR   \| J00-J06 \| Acute upper respiratory infections \| \| --- \| --- \| \| J09-J18 \| Influenza and pneumonia \| \| J20-J22 \| Other acute lower respiratory infections \| \| J30-J39 \| Other diseases of upper respiratory tract \| \| J40-J47 \| Chronic lower respiratory diseases \| \| J60-J70 \| Lung diseases due to external agents \| \| J80-J84 \| Other respiratory diseases principally affecting the interstitium \| \| J85-J86 \| Suppurative and necrotic conditions of lower respiratory tract \| \| J90-J94 \| Other diseases of pleura \| \| J95-J99 \| Other diseases of the respiratory system \| |
| RSV-related rehospitalization | All hospitalizations in PMSI with any of the following diagnosis coded as DP/DR   \| J121 \| RSV pneumonia \| \| --- \| --- \| \| J205 \| Acute bronchitis due to RSV \| \| J210 \| Acute bronchiolitis due to RSV \| |

Supplementary Table 6: Sensitivity analyses on non-specific codes

|  | RSV-specific codes | | | | | RSV non-specific codes | | | | | Number of stays  2016-2023 |
| --- | --- | --- | --- | --- | --- | --- | --- | --- | --- | --- | --- |
| **ICD-10** | J121 | J205 | J210 | B974 | J208 | | J209 | J218 | J219 | J45 |  |
| **Conservative scenario** | All year | All year | All year | / | / | | / | / | / | / | **169,333** |
| **Seasonal scenario – main analysis** | **All year** | **All year** | **All year** | **Season OCT-MAR** | **Season OCT-MAR** | | **Season OCT-MAR** | **Season OCT-MAR** | **Season OCT-MAR** | **Season OCT-MAR** | **293,662** |
| **Annual scenario** | All year | All year | All year | All year | All year | | All year | All year | All year | All year | **418,683** |

J121 (RSV pneumonia), J205 (Acute bronchitis due to RSV), J210 (Acute bronchiolitis due to RSV), B974 (RSV as cause of other diseases), J208 (Acute bronchitis Other organisms), J209 (Acute bronchitis, NS), J218 (Acute bronchiolitis Other organisms), J219 (Acute bronchiolitis, NS) J45 (Asthma)

Supplementary Table 7: Main characteristics by coding scenario

|  | Conservative scenario | Seasonal scenario – main analysis | Annual scenario |
| --- | --- | --- | --- |
| Proportion of females (%) | 44.9% | 42.1% | 41.2% |
| Median (Q1-Q3) age, months | 2.6 (1.3 – 4.9) | 3.3 (1.6 – 6.1) | 3.5 (1.5 – 6.4) |

## Supplementary Figures

Supplementary Figure 1: Codes and algorithms on RSV-related hospitalizations in the literature


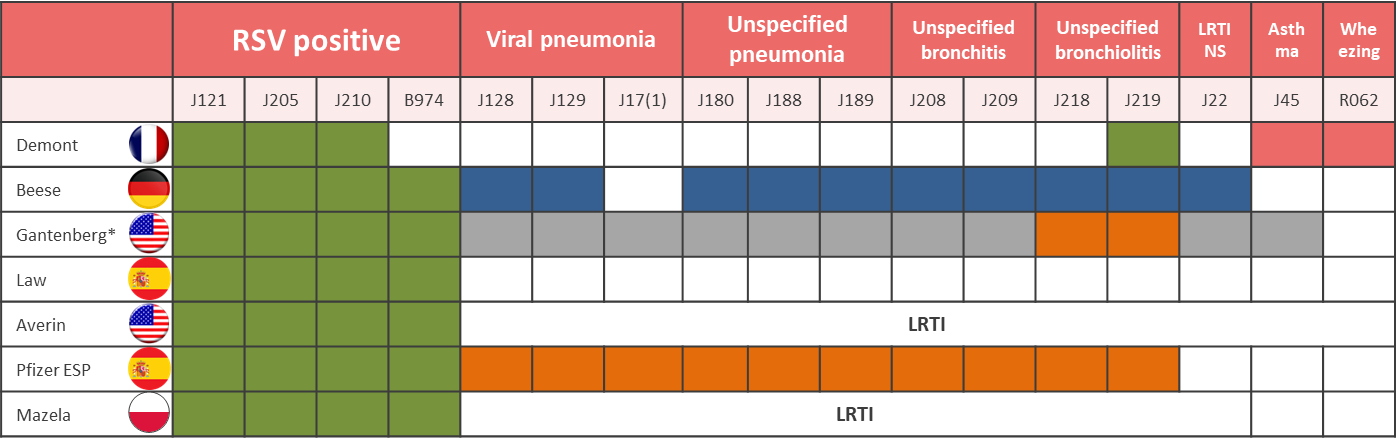


Colors reflect the different coding strategies in each study:

Green: code is looked for through the entire year; Blue: code is looked for only during epidemic season (OCT-MAR); Orange: code is looked for as a sensitivity analysis; Grey: Code is looked for if associated with a RSV positive code; LRTI: lower respiratory tract infection – looked for as sensitivity analyses

References:

1. Demont C, Petrica N, Bardoulat I, Duret S, Watier L, Chosidow A, et al. Economic and disease burden of RSV-associated hospitalizations in young children in France, from 2010 through 2018. BMC Infect Dis. 2021 Aug 2;21(1):730.

2. Beese C, Bayer L, Huebbe B, Riedel J, Melnik S, Brestrich G, et al. Clinical and economic burden of lower respiratory tract infection due to respiratory syncytial virus in young children in Germany [Internet]. medRxiv; 2024 [cited 2024 June 26]. p. 2024.02.12.24302675. Available from: https://www.medrxiv.org/content/10.1101/2024.02.12.24302675v1

3. Gantenberg JR, van Aalst R, Zimmerman N, Limone B, Chaves SS, La Via WV, et al. Medically Attended Illness due to Respiratory Syncytial Virus Infection Among Infants Born in the United States Between 2016 and 2020. J Infect Dis. 2022 Aug 15;226(Suppl 2):S164–74.

4. Averin A, Huebbe B, Atwood M, Bayer LJ, Lade C, von Eiff C, et al. Cost-effectiveness of bivalent respiratory syncytial virus prefusion F vaccine for prevention of respiratory syncytial virus among older adults in Germany. Expert Rev Vaccines. 2025 Dec;24(1):1–10.

5. Mazela J, Jackowska T, Czech M, Helwich E, Martyn O, Aleksiejuk P, et al. Epidemiology of Respiratory Syncytial Virus Hospitalizations in Poland: An Analysis from 2015 to 2023 Covering the Entire Polish Population of Children Aged under Five Years. Viruses. 2024 May;16(5):704.

*Law et al, and Pfizer ESP et al. unpublished data*

Supplementary Figure 2: monthly distribution of RSV hospitalizations by age class


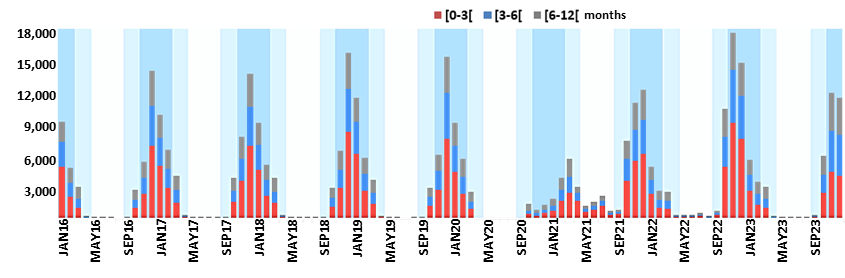


Nirsevimab was first available for infants in France in September 2023, for infants in their first epidemic season. Because of supply shortages, not all infants could be immunized during the 2023-2024 season.

White background: low circulation period (May to August)

Light blue background: transitional periods (September-October and March-April)

Dark blue background: epidemic peak (November to February)

## Abbreviations

DRG: diagnosis-related group

ICD-10 : international classification of diseases – 10^th^ revision

LRTI: lower respiratory tract infection

RSV: respiratory syncytial virus
